# Differential regional expression patterns of α-synuclein, TNF-α, and IL-1β; and variable status of dopaminergic neurotoxicity in mouse brain after Paraquat treatment

**DOI:** 10.1186/1742-2094-8-163

**Published:** 2011-11-24

**Authors:** Soham Mitra, Nilkanta Chakrabarti, Arindam Bhattacharyya

**Affiliations:** 1 Immunology Lab, Department of Zoology, University of Calcutta, 35, Balygunge Circular Road. Kolkata-700019, India; 2 Department of Physiology, University of Calcutta, 92, APC Ray Road, Kolkata-700009, India

**Keywords:** Paraquat, α-synuclein, tyrosine hydroxylase, tumor necrosis factor-α, interleukin-1β, substantia nigra, frontal cortex, hippocampus

## Abstract

**Background:**

Paraquat (1, 1-dimethyl-4, 4-bipyridium dichloride; PQ) causes neurotoxicity, especially dopaminergic neurotoxicity, and is a supposed risk factor for Parkinson's disease (PD). However, the cellular and molecular mechanisms of PQ-induced neurodegeneration are far from clear. Previous studies have shown that PQ induces neuroinflammation and dopaminergic cell loss, but the prime cause of those events is still in debate.

**Methods:**

We examined the neuropathological effects of PQ not only in substantia nigra (SN) but also in frontal cortex (FC) and hippocampus of the progressive mouse (adult Swiss albino) model of PD-like neurodegeneration, using immunohistochemistry, western blots, and histological and biochemical analyses.

**Results:**

PQ caused differential patterns of changes in cellular morphology and expression of proteins related to PD and neuroinflammation in the three regions examined (SN, FC and hippocampus). Coincident with behavioral impairment and brain-specific ROS generation, there was differential immunolocalization and decreased expression levels of tyrosine hydroxylase (TH) in the three regions, whereas α-synuclein immunopositivity increased in hippocampus, increased in FC and decreased in SN. PQ-induced neuroinflammation was characterized by area-specific changes in localization and appearances of microglial cells with or without activation and increment in expression patterns of tumor necrosis factor-α in the three regions of mouse brain. Expression of interleukin-1β was increased in FC and hippocampus but not significantly changed in SN.

**Conclusion:**

The present study demonstrates that PQ induces ROS production and differential α-synuclein expression that promotes neuroinflammation in microglia-dependent or -independent manners, and produces different patterns of dopaminergic neurotoxicity in three different regions of mouse brain.

## Background

Several studies in rodent models have indicated that Paraquat (1, 1-dimethyl-4, 4-bipyridium dichloride; PQ) an environmental herbicide/pesticide, causes neurotoxicity through the generation of reactive oxygen species (ROS) and formation of apoptosis-related molecules. PQ promotes intracellular generation of ROS via three distinct pathways: (1) reduction of PQ by NADPH-cytochrome P450 reductase and a subsequent redox cycle with involvement of super oxide dismutase (SOD) and glutathione pools, (2) inhibition of mitochondrial electron transport chain, and (3) interaction with other enzymes such as nitric oxide synthase (cytosolic), NADPH oxidase (plasma membrane), thioredoxin reductase (cytosolic form, Trx 1), and xanthine oxidase [1]. PQ-induced oxidative stress has been reported to be linked to endoplasmic reticulum stress-signaling pathways and subsequent formation of caspase-dependent apoptosis-related molecules [2,3]. PQ has also been shown to induce neuronal oxidative stress through activation of glial cells [4]. However the exact mechanism of neuronal cell death after PQ administration in rodent models is far from clear. Although carrier-mediated (neutral amino acid transporter carriers, such as LAT-1, which transports L-valine and L-phenylalanine) transport of PQ across the blood-brain barrier (BBB) has been reported in rodent studies [5,6], there is controversy regarding the entry of PQ through BBB, the cellular metabolism of PQ, and the mechanism of its toxicity in brain of non-human primates and human beings  [4,7].

Because of its close structural similarity to 1-methyl-4-phenylpyridinium (MPP+, the active metabolite form of MPTP), Paraquat has been suggested to be a risk factor for PD. Systemic administration of Paraquat to adult mice results in a significant decrease in substantia nigra dopaminergic neurons, a decline in striatal dopamine nerve terminal density, and a neurobehavioral syndrome characterized by reduced ambulatory activity. Prolonged exposure to paraquat leads to a remarkable accumulation of *α*-synuclein-like aggregates in neurons of the substantia nigra pars compacta in mice [8].

PQ-induced dopaminergic neuronal cell death in the substantia nigra (SN) has been found to be linked with aggregation of α-synuclein, in addition to mitochondrial dysfunction and oxidative stress. PQ induces α-synuclein aggregation through protein up-regulation [9,10]. PQ-induced oxidative stress could facilitate α-synuclein association by altering the biophysical properties of the protein, by proteosomal dysfunction, and/or by impairing mechanisms of protein degradation within neurons [4,9,11]. In the Paraquat-induced mouse model of PD, microglial activation and pesticide exposure act synergistically, and the susceptibility of dopaminergic neurons to toxic injury is dramatically exacerbated by underlying inflammatory processes [12]. PQ induces neuroinflammation and microglial activation indirectly through factors released from neurons or astrocytes [13]. PQ induces nigral astrocytosis and microgliosis, the latter showing a reactive phenotype with increased numbers of macrophage antigen complex-1-immunoreactive cells (a marker for activated microglial cells) [14,15]. Dopaminergic neurons in the substantia nigra and ventral tegmental area have different susceptibilities to damage by PQ toxicity [16], and major unanswered questions include whether the protein aggregates cause the selective loss of dopaminergic neurons in the substantia nigra that underlies the clinical symptoms and whether neuroinflammation is a consequence or a cause of nigral cell loss [17].

Apart from SN, PQ can also damage hippocampal neurons of mouse brain through oxidative stress-induced mitochondrial dysfunction [18]. PQ also induces cell loss in locus coeruleus, in the area in which catecholaminergic neurons are located [19]. *In vitro *studies have shown that PQ induces apoptosis of cultured rat cortical cells [20]. It is not clear whether PQ-induced dopaminergic cell death is selective or if other cell types are similarly affected [18,4,21] in other regions of brain such as frontal cortex (which is primarily responsible for cognitive and motor responses) and hippocampus (which is primarily responsible for learning, cognition and memory).

Studies with rodent models have suggested that PQ is a potential risk factor for Parkinson's disease (PD). PQ-induced neurotoxicity and PD pathology show molecular similarities including protein aggregation [22], neuroinflammation [23], oxidative stress [24], mitochondrial dysfunction [25]and caspase activation [26]. However, a role for PQ in causing Parkinsonism in non-human primates and human beings is uncertain due to a lack of experimental and clinical evidence.

The exact mechanism of PQ-induced neurotoxicity is, therefore, still in debate. An understanding of the molecular basis of PQ-induced neurotoxicity could provide valuable insights into neurodegenerative processes in mammalian brain. In the present study, we sought to define PQ-induced changes in molecular events associated with dopaminergic neurodegeneration in three regions of brain: SN, hippocampus, and frontal cortex (FC). Although PQ toxicity causes dopaminergic cell death in SN, the site of origin of dopaminergic innervation in brain, an effect of PQ on dopaminergic neuronal processes in hippocampus and FC has not been established. PQ toxicity and PD-like motor dysfunction/cognitive impairment both are accompanied by neuronal damage in hippocampus and frontal cortex. The main objective of our present study was to assess possible molecular links between PQ-induced dopaminergic neurotoxicity, alteration of α-synuclein status, and microglial activation in three regions of brain. Dopaminergic neurotoxicity was determined using the neuronal markers FOX-3, tyrosine hydroxylase (TH; the rate-limiting enzyme of DOPA synthesis) and DOPA decarboxylase (the enzyme that catalyzes decarboxylation of L-dopa to dopamine). The microglial marker Iba-1, the microglial activation marker Mac-1, the histological feature of microglial aggregation, and microglial expression of the cytokines interlukin-1ß (IL-1ß) and tumor necrosis factor- α (TNF-α) were determined in the three brain regions. The study was extended with tocopherol (an ROS scavenger) supplementation followed by PQ treatment to assess PQ-induced ROS generation and its possible impact on alterations in α-synuclein status and microglial activation.

## Methods

### Materials

Paraquat dichloride (PQ; 1,1'-Dimethyl-4,4'-bipyridinium dichloride hydrate) and α-tocopherol were purchased from Sigma Aldrich, Inc. (St. Louis, MO). Among the primary antibodies used, anti-tyrosine hydroxylase mouse monoclonal antibody was purchased from Calbiochem (EMD4Biosciences, NJ, USA); rabbit polyclonal anti-TNF-α and anti-IL-1β, and mouse monoclonal anti-α-synuclein were procured from Cell Signaling Technology, Inc. (Danvers, MA, USA); mouse monoclonal anti-FOX3, anti-DOPA decarboxylase, goat polyclonal anti-Iba 1, and rabbit polyclonal anti-Mac1 were procured from Abcam plc (Cambridge, UK). The secondary antibodies goat anti-rabbit IgG--HRP (horseradish peroxidase) and rabbit anti-mouse IgG--HRP, and a DAB developing system (for immunohistochemistry) were purchased from Bangalore GeNei Pvt. Ltd. (Bangalore, India). FITC-conjugated secondary mouse anti-goat antibody was purchased from Santa Cruz biotechnologies (DA, USA). Hematoxyline and eosin were obtained from Merck Specialties Private Limited (Mumbai, India) and the remaining chemicals were purchased in analytical grade of highest purity (India).

### Animals

Adult male Swiss albino mice weighing ~28 gm each (22-24 weeks of age; five mice in each group) were obtained from the National Institute of Nutrition (Hyderabad, India). All animals were housed individually for at least one week prior to experiments in an animal facility (maintained at 25 [ ± 2]°C with 55 [ ± 5]% relative humidity and 12-hr light/dark cycle) located at the Animal Housing Unit in the Department of Zoology, University of Calcutta. All animals were provided rodent chow obtained from National Institute of Nutrition (Hyderabad, India) and filtered water *ad libitum*. All animal experiments were performed following "Principles of laboratory animal care" (NIH publication No. 85- 23, revised in 1985) as well as specific Indian laws on "Protection of Animals" under the provision of authorized investigators. The protocols were approved by the Institutional Animal Ethics Committee at the University of Calcutta.

### PQ administration and supplementation with α-tocopherol

A total of 50 mice received intraperitoneal (i.p) injections of PQ at different concentrations [5, 10, 20, 40, 80 mg/kg body weight (b.w.)] in a total volume of 0.2 ml, twice a week for four consecutive weeks to determine the LD_50 _dose (n = 10 for each dose of PQ). Thereafter, based on this data, new sets of mice were provided with sublethal doses (10 mg PQ/kg b.w.) twice a week for 4 weeks. Mice were randomly divided into 4 groups comprising (A) vehicle/saline (0.9% NaCl)-treated control (n = 6), (B) PQ-treated (n = 6), (C) α-tocopherol--supplemented. PQ-treated (n = 6), and (D) α-tocopherol-supplemented, vehicle/saline-treated controls (n = 6) for the experiments to be performed.

α-Tocopherol was supplemented to mice that had been treated previously with PQ, 10 mg/kg b.w. for four weeks. α-Tocopherol was injected intraperitonealy at 20 mg/kg b.w. for five consecutive days after the last dose of PQ (up to day six) and sacrificed thereafter on day seven.

### General health and gross motor functions assessment

Mice were observed twice daily during the first week of injections (10 mg PQ/kg b.w. for each mouse) and daily thereafter. General health and gross motor functions were assessed by observing in-cage behavior and during brief gentle handling to check for rigidity (hunched posture and increased tail tone), bradykinesia (slowed movement and/or absence of rearing), dystonia (clenched paws), autonomic signs (piloerection) and stopped movement (akynesia). Additionally, body weights were checked prior to and each week after PQ administration. Simple behavioral tests in control and PQ-treated mice were performed three days after the last injection. Asymmetry in body posture and gait abnormalities were tested with the curling test and the footprint test, respectively. The curling test evaluates any asymmetry in body posture [27]. The mice were lifted gently 2-3 cm above the bedding for 5 seconds and any ipsilateral deviation from its vertical body axis of 10° or greater was recorded. The test was repeated three times for each animal.

For the footprint test mice were placed in a 5-cm-wide, 55-cm-long corridor. The floor of this corridor was covered with white absorbant paper. The animals were first trained to pass straight forward through the corridor. After this training, the paws were colored with different colors (red for the forepaws and violet for the hindpaws), and the mice were then placed into the corridor. Step frequency and stride length were determined with the program Footprints version 1.22 [27].

### Tissue handling

On the 7^th ^day after the final dosing with PQ, mice in each group were euthanized by over-dose of sodium thiopentone (Mancure Drugs Private Ltd., Mumbai, India) and brain tissues harvested for analyses as described in the assays below. The animals used in behavioral studies were same animals that were sacrificed thereafter for brain tissue analysis.

### Histological analysis

Animals were deeply anesthetized by overdose of sodium thiopentone (Mancure Drugs Private Ltd., Mumbai, India) and were sacrificed by decapitation. Brains were removed immediately and washed in ice-cold phosphate-buffered saline (PBS, pH 7.4). Then, the tissues were cut into two equal halves along the longitudinal fissure. The tissues were fixed for 24 hours in buffered formaldehyde solution (10% in PBS) at room temperature, dehydrated by graded ethanols (50-100%) and embedded in paraffin (Merck, solidification point 60--62°C). Tissue sections (thickness 5 μm) were then deparaffinized with xylene, rehydrated with graded alcohols (100%-50% ethanol), stained with eosin/haematoxylin (Merck, Mumbai, India) and mounted in DPX resin (Merck, Mumbai, India). Images were captured using an Olympus BX51 microscope attached to an Olympus DP70 camera (U-TVO 63 × C; Olympus Corp., Tokyo, Japan) having both 40× and 100× (wide zoom) lenses.

### Immunohistochemistry

Sagittal brain sections (5 µm thick) were cut from paraffin-embedded brain tissue and mounted on positively-charged Super frost slides (Export Mengel CF, Menzel, Braunschweig, Germany). Tissues were deparaffinized, dehydrated through graded alcohols, and then endogenous peroxidase was quenched in a 3% hydrogen peroxide solution for 20 minute at room temperature. Background staining was then inhibited with 5% bovine serum albumin [BSA] (Sisco Research Laboratories Pvt. Ltd. [SRL], Mumbai, India) for 30 minutes at room temperature to avoid nonspecific binding of IgG. Excess liquid was drained and the sections were incubated in a humid chamber overnight at 4°C with primary antibodies (diluted 1:50 in solution containing 5% BSA). The following specific primary antibodies were used for separate cases:,anti-IL-1β (mouse polyclonal), anti-TNF-α (mouse polyclonal), and anti-α-synuclein (mouse monoclonal) (Cell Signaling Technology, Inc. [Danvers, MA, USA]), as well as anti-tyrosine hydroxylase (mouse monoclonal) (Calbiochem [EMD4Biosciences, NJ, USA]) for positive controls (data not shown). After three washes in PBS-T, sections were sequentially incubated in horseradish peroxidase (HRP)-conjugated anti-sera specific for those antigens and were diluted at a 1:30 ratio in Tris-buffered saline containing 0.3% Triton-X and 0.5% blocking agent for 2 hours at room temperature. Immunoreactive complexes were then detected using a DAB system (Merck Specialties). Sections were then counterstained briefly in hematoxylin, dehydrated through graded alcohols (70%, 95%, 100%), cleared in xylenes, and coverslipped with DPX mounting medium. Slides that received no primary antibody served as negative controls. Images were captured using a U-TVO 63 × C microscope (Olympus Corp., Tokyo, Japan) having both 40× and 100× (wide zoom) lenses.

### Preparation of cell lysates

Different brain regions -- hippocampus, frontal cortex, and SN -- were dissected out immediately after dissection of sagittal sections of whole brain [28]. Tissues were homogenized in ice-cold RIPA lysis buffer (150 mM sodium chloride, 1.0% TritonX-100, 50 mM Tris pH 8.0, 0.01% SDS, 0.5% sodium deoxycholate) containing 1 mM PMSF (phenylmethanesulfonylfluoride or phenylmethylsulfonyl fluoride) (SRL, India), 1 μg/ml aprotinin, 1 μg/ml leupeptin and 1 μg/ml pepstatin (Sigma-Aldrich Inc., USA) that were added fresh prior to cell lysis. The samples were sonicated and incubated on ice for 30 min, and centrifuged 3 times at 14,000 rpm for 15 min at 4°C. A portion of the supernatant was reserved for protein determination using the Bradford reagent (Sigma-Aldrich Inc., USA) and subsequent measurement of absorbance was done at 595 nm in a UV-1700 PharmaSpec, Shimadzu spectropho-tometer (Shimadzu Scientific Instruments, Columbia, MD). The remaining supernatant was stored at -20°C.

### Western blot analysis

Tissue lysates were diluted in sample buffer (0.312 mM Tris-HCl [pH 6.8], 50% glycerol, 10% SDS, 25% β-mercaptoethanol, and 0.25% bromophenol blue) at a final protein concentration of 5 μg/μl, and were then boiled at 100°C for 5 minutes. Aliquots of each sample (10 μl containing 50 μg protein) were loaded into dedicated wells of 9-12% polyacrylamide gels and separated by electrophoresis for 3 h at 100 V. Proteins were transferred to polyvinylidene difluoride membrane (Amersham Biosciences, Piscataway, NJ) for 1.5 h at 300 mA. After blocking of nonspecific binding with 5% nonfat dry milk in TBST, the membranes were then probed with the following primary antibodies: anti-tyrosine hydroxylase (mouse monoclonal; 1: 2000 dilution), anti-TNF-α, anti-IL-1β (rabbit polyclonal; 1: 1000 dilution), anti-α-synuclein (mouse monoclonal; 1: 1500 dilution) anti-FOX3, anti-DOPA decarboxylase (mouse monoclonal; 1: 2000 dilution), anti-Iba1 (goat polyclonal; 1: 2500 dilution), anti-Mac1 (rabbit polyclonal; 1: 2000 dilution) and incubated overnight at 4°C. The membranes were washed 3 times with Tris-buffered saline-0.01% (v/v) containing Tween-20 at room temperature for 15 minutes and then incubated with alkaline phosphatase (AP)-conjugated secondary antibodies (anti-rabbit, anti-goat and anti-mouse IgG; diluted 1:1000) with TBST for 2 h at room temperature. The membranes were then developed with NBT/BCIP (nitroblue tetrazolium chloride/5-bromo-4-chloro-3-indolyl-phosphate; Hi-Media, Mumbai, India). Band intensity of the detected protein was measured by densitometry (Gel Doc™ XR+ System, Bio-Rad Laboratories, USA). β-Actin was also analyzed on each membrane for confirmation of gel sample loading (i.e., based on constitutive expression).

### Immunofluorescence

The immunofluorescence procedure was carried out on sections incubated in blocking buffer (0.3-0.5% Triton X-100, 5% BSA in TBS) for 30 min at room temp followed by an overnight incubation with the primary antibody Iba1 (goat polyclonal; 1: 50 dilution) in blocking buffer at 4°C,. After three washes in TBS-T, primary antibody was revealed by incubating the sections in FITC-conjugated anti-sera specific for those antigens and diluted at a 1:30 ratio in Tris-buffered saline containing 0.3% Triton-X and 0.5% blocking agent for 2 hours at room temperature followed by TBS washing. The tissue sections were counterstained with a nuclear counterstain (DAPI by Vector Laboratories Inc. Burlingame, CA, USA) and mounted with DPX resin. Images were captured in a U-TVO 63 × C microscope (Olympus Corp., Tokyo, Japan) having both 40× and 100× (wide zoom) lenses.

### Stereological cell counting

Stereological methods were used for quantification of cells present in stained tissues as previously described [29]. Briefly, tissue was visualized with an Olympus U-TVO 63 × C microscope and Micro Bright Field CX 9000 camera. Cell counting was done for both side of the brain for each animal, and then right and left values were added to generate a total DA substantia nigra neuron count, in a total of five animals per experimental group. The tissue was quantified using optical fractionators from MicroBrightField, with the software Stereo Investigator (Ver.8). Estimated volumes (μm^3^) of TH-negative zones were quantified using the cavalieri method of unbiased stereology in the substantia nigra of every third section. Also, expression of FOX 3-positive cells was quantified in substantia nigra using the cavalieri method. Both immunostains were quantified with a grid spacing of 200 μm using a 2×/0.06 objective. TH-positive cells were also quantified within the area of the substantia nigra pars compacta. The sampling site was customized to count 200 cells per brain, and sampling was done with error coefficients less than 0.07. For counting TH-positive cells the counting frame (CF) was 70 × 70 with a virtual counting grid (CG) of 140 × 140. For FOX3-positive cells CF and CG were the same as for TH-positive cells.

### Microglia-specific silver staining

Microglia-specific silver staining was performed as previously described [30]. Briefly, paraffin-embedded mouse brain sections were deparaffinized with xyline followed by rehydration in a series of graded (100%-50%) ethanols. After washing in distilled water, sections were transferred for silver impregnation in 10% ammoniacal silver nitrate solution for 3-4 seconds. After that, sections were transferred to a 10% formalin solution and then washed with distilled water. The slides were then fixed in 5% sodium thiosulfate solution for 2-5 minutes. Sections were washed and then dehydrated in graded alcohols and finally mounted in DPX (MERCK) for microscopical studies. Images were captured in an Olympus BX51 microscope attached with Olympus DP70 camera (U-TVO 63 × C) (Olympus, USA).

### Preparation of homogenatse for ROS scavenging enzyme assay

A 10% tissue homogenate was prepared in 0.1 M phosphate buffer (pH 7.4) containing 0.1 M KCl. Enzyme assays were performed in the supernatant obtained following centrifugation of the homogenate at 9000 × g for 10 min at 4°C.

## Measurement of catalase activity

Catalase was measured usig the method described by Sinha et al. (1972) [31]. In brief, an assay mixture consisting of 0.01 M phosphate buffer (pH 7.0), 0.2 M hydrogen peroxide and tissue homogenate was incubated at 37°C for 1 min. The reaction was stopped by addition of potassium dichromate (5% w/v) and acetic acid. The remaining hydrogen peroxide was determined by measuring chromium acetate after heating the assay mixtures in a boiling water bath for 15 min. Absorbance was read at 570 nm against control without hydrogen peroxide. Enzymatic activity is expressed as μmol/min/mg protein.

### Estimation of glutathione S-transferase activity

Glutathione S-transferase was assayed spectrophotometrically by the method described by Pabst et al. (1974) [32]with slight modifications. In brief, 150 μl homogenate was mixed with 0.2 M phosphate buffer (pH 6.5). The reaction was initiated by addition of 20 μl 3% CDNB (1 chloro-2,4-dinitrobenzene). Optical density was measured at 340 nm at intervals of 30 s for 3 min, and activity was calculated as nM/min/mg protein.

### Measurement of total cytosolic superoxide dismutase

Superoxide dismutase (SOD) was measured spectrophotometrically at 570 nm using the modified method of Kakkar et al. (1984) [33]. In brief, an assay mixture containing sodium pyrophosphate buffer (pH 8.3, 0.052 M), phenazine methosulfate (186 μM), nitroblue tetrazolium (300 μM), NADH (780 μM) and appropriately diluted enzyme in a total volume of 3 ml was incubated at 37°C for 90 s. The reaction was stopped by addition of glacial acetic acid. The reaction mixture was mixed vigorously by adding n-butanol and was allowed to stand for 10 min before collection of the butanol layer. The intensity of chromogen in the butanol was measured at 560 nm. SOD activity was calculated as μmol/min/mg protein.

### Statistical analysis

Values between groups were analyzed using single way ANOVA. All values are shown as mean ± SEM, except where otherwise indicated. Data were analyzed and when appropriate, significance of the differences between mean values was determined using Student's t-test. Results were considered significant at p < 0.05.

## Results

### Effect of PQ on mice survival

**Figure 1:**
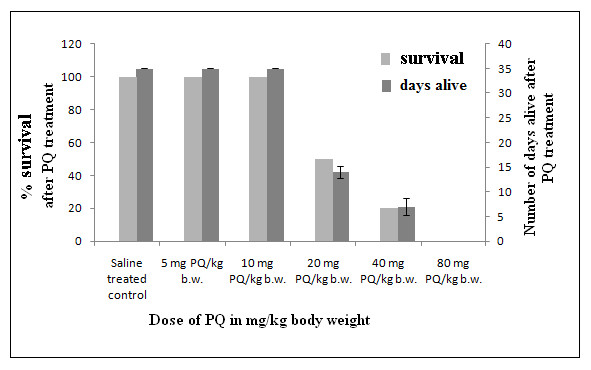
**Dose-dependent effects of PQ on survival of mice and identification of a sublethal dose of PQ**. Survival of mice indicates number of animals alive after PQ treatment. Intraperitoneal injection of PQ with various doses (5, 10, 20, 40, 80 mg/kg b.w.) caused death of animals at doses of 20, 40 and 80 mg/kg b.w. of PQ. Animals treated with PQ at doses of 5 and 10 mg/kg b.w. remained alive during the entire treatment period, and were sacrificed after the final treatment as described in Methods. Animals treated with 80 mg PQ/kg b.w. died within 8 hours of the 1^st ^dose. Experiments were performed with 10 animals in each group. Error bars represent animals that were alive on different days after treatment of PQ with doses of 20 mg/kg b.w. and 40 mg/kg b.w.

To assess a permissible dose, different doses of PQ (5, 10 [34], 20 [35], 40 [36]and 80 mg PQ/kg b.w.) were administered to mice twice a week over a 4-week period based on previous reports. Experimental animals treated with PQ at a dose of 10 mg/kg b.w. did not show any mortality through day 35, when they were sacrificed. Survival decreased with greater increments of dosage: 50% and 80% of animals died at doses of 20 mg/kg b.w. (within the first 15 days of treatment, after a total of 4 or 5 doses per recipient) and 40 mg PQ/kg b.w. (within the first 8 days of treatment, after 1 or 2 doses per recipient) treatments, respectively, and animals that were treated with PQ at a concentration of 80 mg/kg b.w. died after the first treatment. Therefore, further experiments were performed using a dose of 10 mg PQ/kg b.w. (a sublethal dose) (Figure 1).

### Behavioral changes observed in PQ-treated (10 mg/kg b.w.) mice

**Figure 2:**
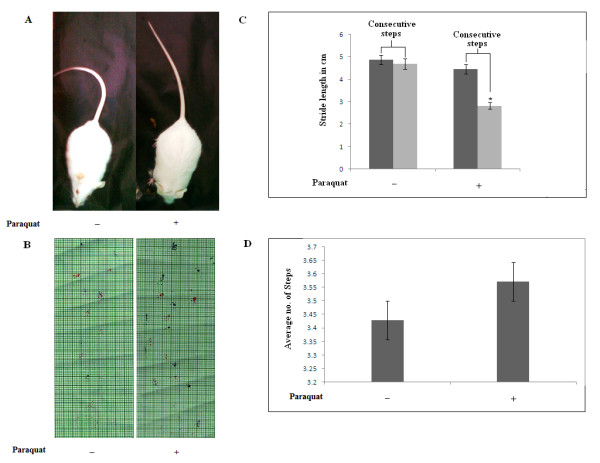
**Symptoms of motor dysfunction in mice treated with a sublethal dose (10 mg/kg b.w.) of PQ**. PQ caused severe postural instability (A) and gait impairment (B). **(A): **The curling test evaluated asymmetry in body posture. There was severe hunched-back deviation from the vertical body axis in PQ-treated animals. **(B): **Representative walking footprint patterns displayed irregular stride length in consecutive steps in treated animals. Control animals followed a straight walking pathway, whereas treated animals deviated from the normal walking pathway within a confined area. **(C): **Bar graph indicates differential stride length of consecutive steps in PQ-treated animals compared to controls. **(D): **There were no significant differences in average number of steps (step frequency) for PQ-treated animals compared to vehicle-treated controls. Values in figure C and D represent mean ± SEM (n = 3). Asterisk (*) indicates a significant change in stride length, p < 0.05 (Student's t-test).

Motor performance deteriorated greatly in the treatment group (10 mg/kg b.w.) of animals after two weeks of treatment, and this deterioration was progressive over the next several days. Several symptoms appeared, including outward curvature of the spine (i.e. hunched posture like kyphosis), piloerection, and twisting with repetitive movement by involuntary contractions of muscle, called dystonia. With additional days of PQ treatment, mice showed reduction of progressive and spontaneous motor activity, trembling, drooling, lassitude, clumsy limb activity and even inactivity. After administration of the final dose and before the day of sacrifice (day 35), the treatment group of mice showed trembling, hypolocomotion, gazing, polypnoea, arched back, stretched hind limbs, unstable gait, tail hyperextension and irritability. To address the posture and walking difficulties associated with neurodegeneration, simple behavioral tests were performed three days after the last injection in control and PQ-treated mice (Figure 2). It is clear that mice treated with 10 mg/kg b.w. PQ exhibited severe postural instability, consistent with a unilateral lesion. All of the mice in this group displayed ipsilateral curling (Figure 2A). No asymmetry was observed in the saline-treated control groups. Treated mice showed gait impairment assessed by footprint patterns (Figure 2B), in particular stride length and step frequency (Figure 2C and 2D respectively). Irregular stride length in consecutive steps was noted in PQ-treated animals.

### ROS-scavenging enzymes (catalase, GST and SOD) activity increased in all three regions of brain with sublethal as well as lethal doses of PQ

It is evident that PQ causes neurotoxicity through the generation of reactive oxygen species (ROS). Therefore, to assess whether increased ROS levels are involved in PQ-mediated neurotoxicity we assayed different ROS scavenging enzymes -- catalase, SOD and GST -- in three different brain regions of PQ-treated animals and saline-treated controls.

**Figure 3:**
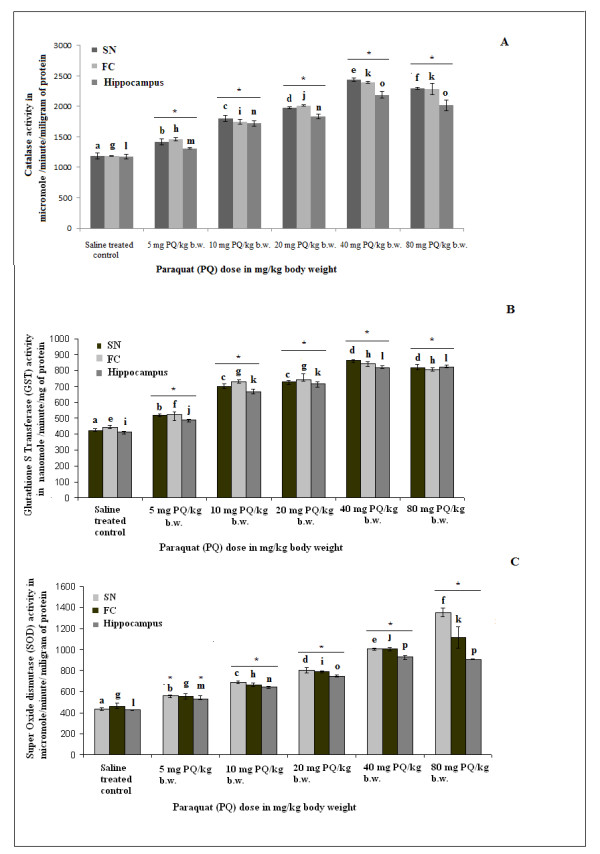
**Dose-dependent rise in activity of ROS-scavenging enzymes with PQ treatment in three regions of mouse brain**. PQ treatment with different doses of PQ (5, 10, 20, 40, 80 mg/kg b.w.) enhanced the activities of catalase (A), GST (B) and SOD (C) in SN, hippocampus and FC of mouse brain. The activities of the enzymes indicate the status of ROS in tissues as described in the Results and Discussion sections. Enzyme activity is presented as μmole/min/mg protein (for catalase and SOD activity) or as nmole/min/mg protein (for GST activity) as indicated in the figures. Asterisks (*) indicate significant differences (p < 0.05, ANOVA) in values for different doses compared to controls. The same letter indicates nonsignificant differences and different letters indicate significant differences (p < 0.05, Student's t-test) between groups.

**Figure 4:**
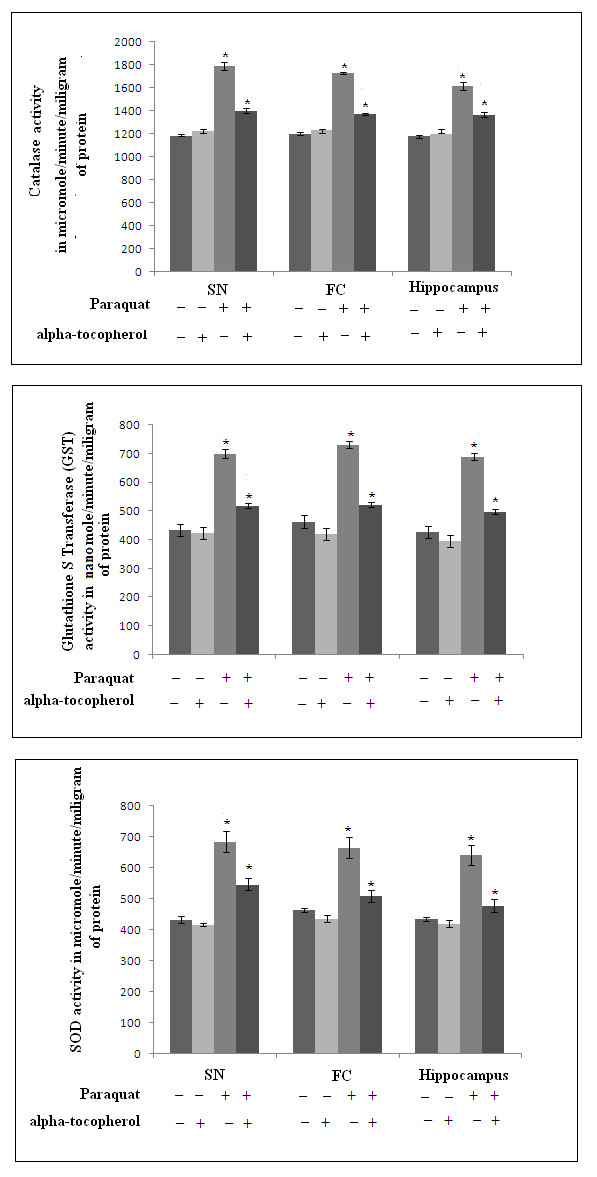
**Tocopherol supplementation reduces PQ-induced activation of ROS-scavenging enzymes**. The activities of catalase, GST and SOD (A, B and C) were measured in SN, FC and hippocampus of mouse brain after PQ treatment. PQ treatment increased enzyme activities significantly compared to saline-treated controls. The values of enzyme activities after PQ treatment followed by tocopherol supplementation remained significantly lower than PQ-treated values, and were higher than saline-treated control values. Tocopherol supplementation did not alter enzyme activity compared to control values.

Significant enhancements in catalase, GST and SOD activities in all three areas of brain were observed in PQ-treated animals (10 mg/kg b.w. dose), compared to control animals. With other, lethal doses (20-80 mg PQ/kg b.w.) catalase, GST and SOD activity was also increased progressively with increment of dose over saline-treated controls (Figure 3A, B and 3C, respectively). Supplementation with α-tocopherol in PQ-treated mice decreased catalase, GST and SOD activities significantly in all three regions of brain compared to mice treated only with PQ (Figure 4A, B and 4C, respectively).

### ROS scavenging enzyme activity in peripheral organs did not change with sublethal doses but increased progressively with increments of lethal doses of PQ

**Figure 5:**
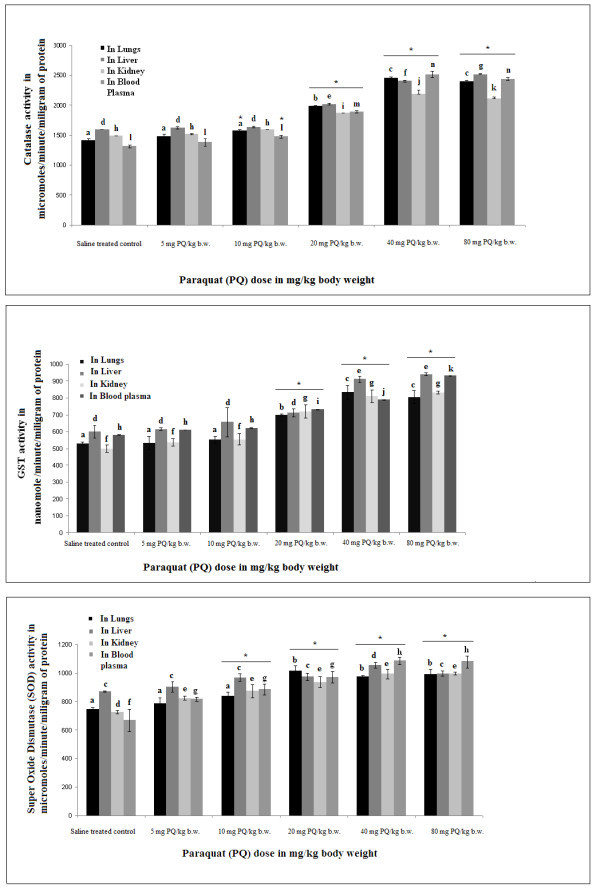
**Dose-dependent effects of PQ on ROS-scavenging enzymes in blood plasma and peripheral tissues**. Lethal doses of PQ (20, 40, 80 mg/kg b.w.) enhanced the activities of catalase (A), GST (B) and SOD (C) in blood plasma and peripheral tissues (lung, liver and kidney) of mice. The activities of these enzymes indicate the status of ROS in tissues and blood plasma. Enzyme activity is presented as μmole/min/mg protein (for catalase and SOD activity) or as nmole/min/mg protein (for GST activity) as indicated in the figures. Asterisks (*) indicate significant differences (p < 0.05, ANOVA) in values for different doses compared to controls. The same letter indicates nonsignificant differences and different letters indicate significant differences (p < 0.05, student t-test) between groups.

Our sublethal dose treatment (10 mg PQ/kg b.w.) produced substantially higher activity level of ROS-scavenging enzymes, indicating high levels of ROS production in all three regions of brain. To assess whether this sublethal dose produces high peripheral level of ROS that might damage peripheral tissues, we have estimated catalase, GST and SOD activities in peripheral tissues like lung, kidney, liver and blood plasma. There were significantly increased activities of these ROS scavenging enzymes in lungs, kidney, liver and blood plasma at all three lethal doses but not with our experimental sublethal dose (Figure 5A, B and 5C).

### Tyrosine hydroxylase- and FOX 3-positive cell numbers showed greatest decrease in SN after sublethal doses of PQ, but not after lower sublethal or higher lethal doses

**Figure 6:**
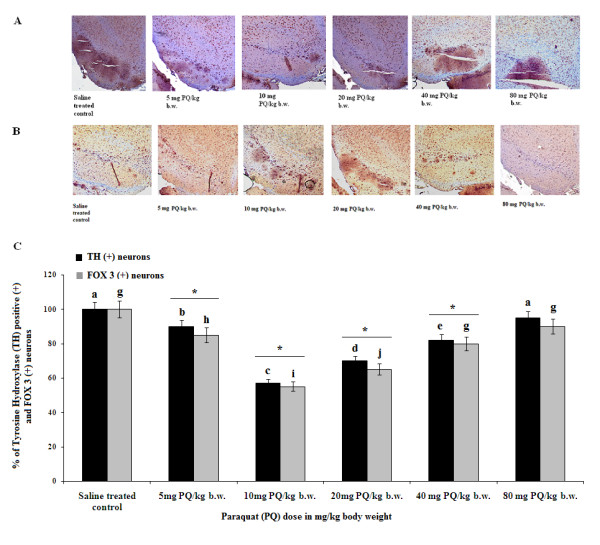
**Dose-dependent variable effects of PQ on dopaminergic cell counts in SN**. Immunopositivity for FOX3 (A) and TH (B) represent dopaminergic neuronal cells in SN during treatment of mice with different doses of PQ. Different doses of PQ (5, 10, 20, 40 and 80 mg/kg b.w.) decreased cell counts variably in SN with a maximum observed effect at a PQ dose of 10 mg/kg b.w. (a sublethal dose) (C). Details of immunohistochemistry and cell counting are described in Methods. Values are presented as mean ± SEM (n = 3). Asterisks (*) indicate significant differences (p < 0.05, ANOVA) in cell counts for different doses compared to controls. The same letter indicates nonsignificant differences and different letters indicate significant differences (p < 0.05, Student's t-test) between groups.

TH-positive and FOX3-positive neuronal cell numbers decreased most significantly in SN of PQ (10 mg/kg b.w.)-treated animals compared to controls (Figure 6A, B and 6C). With other lethal and lower sublethal doses, cell counts varied with significant decreases or no changes compared to controls due to variable PQ exposure duration. With an 80-mg/kg b.w. dose there were no changes in cell numbers compare to control due to the fact that these mice received only a single exposure to PQ.

### Effects of PQ on changes in cellular morphology and histology in SN, FC and hippocampus

Hematoxylin and eosin staining was performed on sagittal sections of whole brain to observe histological and morphological changes in SN, FC and hippocampus of PQ-treated mice.

#### Pyknotic nuclei appeared in all three regions of brain

**Figure 7:**
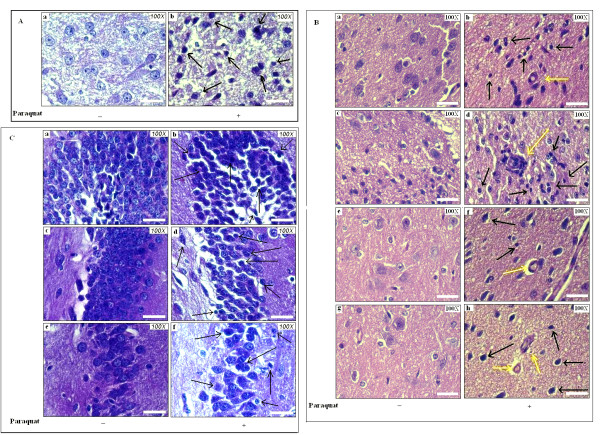
**Histological and morphological changes in three regions of brain after PQ (10 mg/kg b.w.) treatment**. Morphological alterations in cells were observed by H&E staining in SN, FC and hippocampus of PQ-treated animal brains compared to controls. The detailed procedure for staining is described in Methods. **(A): **Formation of pyknotic nuclei (black arrows) is found in SN of PQ-treated mouse brain, but not in vehicle-treated controls. **(B): **Pyknotic nuclei (black arrows) and Lewy body-like structures (yellow arrows) appeared in FC of PQ-treated mouse brain (b, d, f and h) but not in controls (a, c, e and g respectively). Lewy body-like structures of different shapes with acidophilic central cores were found in four regions of frontal cortex. Lewy body-like structures were round-to-oval-shaped (b, h), or irregular-to-pear-shaped (d, f). **(C): **Pyknotic nuclei were observed in the hippocampal regions dentate gyrus, CA3 and CA1 in PQ-treated animals (b, d, f respectively) but not in control brain (a, c, e respectively). Magnification is 100× as indicated; scale bars = 10µm.

As there was a significant decrease in FOX 3- and TH-positive cell counts in SN, we also evaluated PQ-induced cellular changes in SN as well as in the other two brain regions examined. Many pyknotic nuclei were found in SN (Figure 7A). Frontal cortical areas also contained many pyknotic nuclei (Figure 7B). Loss of neuronal cells and formation of pyknotic nuclei appeared in different areas, including hippocampal dentate gyrus, CA3 and CA1 regions (Figure 7C) in PQ-treated mouse brain compared to controls.

#### Lewy body-like formation in FC

Different types of Lewy body-like formations were found in FC of PQ-treated mouse brain in the present study. Rounded and oval-shaped Lewy body-like structures with acidophilic central cores were present in FC (Figure 7B b, h). Irregular-shaped Lewy-like inclusions between cellular processes appeared in pigmented cells of FC. At the margin of dead cells, several Lewy bodies were also observed (Figure 7B d). Pear-shaped Lewy inclusions were also observed in FC (Figure 7B f). However, Lewy bodies did not appear in FC of vehicle-treated control mouse brains (Figure 7B a, c, e and g respectively).

### Differential expression and localization of tyrosine hydroxylase (TH) observed in SN, FC and hippocampus of PQ-treated (10 mg/kg b.w.) mouse brain

**Figure 8:**
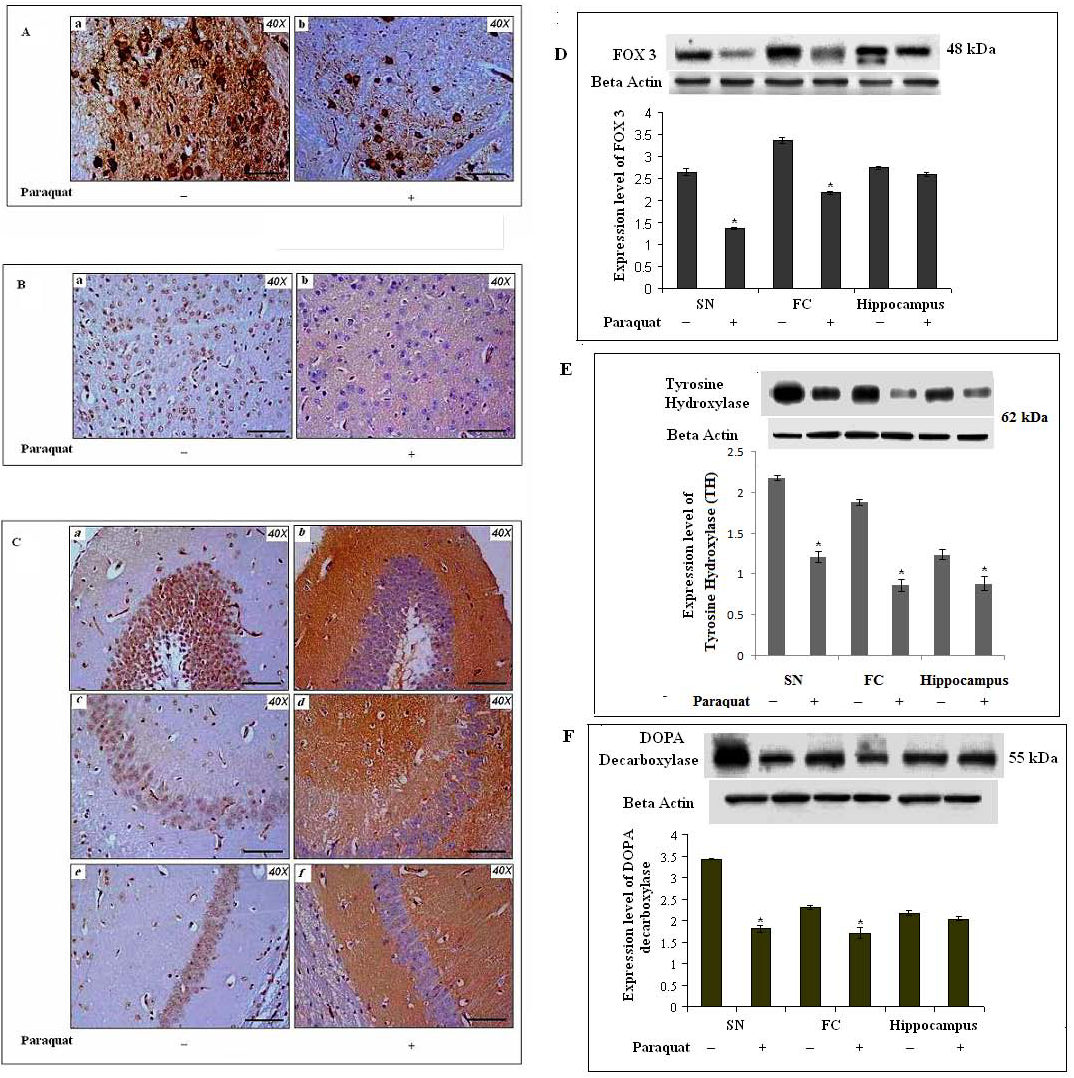
**Differential patterns of immunoreactivity of dopaminergic neuronal markers in brain after PQ (10 mg/kg b.w.) treatment**. Immunoreactive localization of TH was observed in SN, FC and different parts of the hippocampus (A, B and C, respectively). Densitometric analysis of western blots for FOX3, TH and DOPA decarboxylase (D, E and F respectively) was performed to assess the status of dopaminergic neurons in SN, FC and hippocampus. **(A): **TH-immunopositive neuronal cells (pigmented) and neurites were decreased in SN of PQ-treated mice (b) compared to controls (a). **(B): **The cellular localization of TH immunoreactivity shifted from nucleated to non-nucleated neuritic areas in FC in PQ-treated animals (b) compared to controls (a). **(C): **TH immunopositivity appeared in non-nucleated neuritic areas of the hippocampus (dentate gyrus, CA3 and CA1) in PQ-treated animals (b, d, f respectively) compared to TH immunolocalization in nucleated areas in controls (a, c and e respectively). **(D and E): **Expression levels of TH decreased in SN, FC and hippocampal areas of PQ-treated mice compared to respective controls (E). PQ treatment caused significant decreases in expression levels of FOX 3 (D) and DOPA decarboxylase (F) in SN and FC, without any changes of those parameters in hippocampus, compared to respective controls. β-Actin was used as a reference control in the western blots. Data are presented as mean ± SEM. (n = 3). Asterisks (*) represent significant differences at a level of p < 0.05 (Student's t-test) for the densitometric analyses. Magnification of IHC images is 40×, and the scale bars = 40µm.

FOX 3-positive and TH-positive neuronal cell counts decreased, and the morphological appearance of cells varied among different regions of brain in PQ-treated mouse brain (as reported in Figure 6 and 7, respectively). As ROS levels increased in all three regions of the brain, we checked the status of the most vulnerable neuronal populations during oxidative stress conditions, i.e, dopaminergic neurons in all three regions of the brain under investigation. Studies with inmmunohistochemistry and western blot techniques indicated differential expression patterns for FOX3, DOPA decarboxylase, and immunolocalization of TH in all three areas of brain (Figure 8). The immunoreactivity of TH shifted from nucleated to non-nucleated areas in hippocampal regions (Figure 8C). Such translocation of TH-immunoreactivity also appeared in FC (Figure 8B) but not in SN (Figure 8A). Western blot analysis indicated that FOX 3 expression levels decreased in SN and in FC without any change in hippocampus of PQ-treated animals compared to controls (Figure 8D). TH immunoreactivity decreased significantly in all three regions of brain in treated animals compared to controls (Figure 8E). The expression level of DOPA decraboxylase (a key enzyme in dopamine biosynthesis) decreased significantly in SN and FC of PQ-treated mouse brain compared to that of non-treated brain, without any change in hippocampus (Figure 8F). Supplementation with α-tocopherol did not result in any significant changes in FOX3, TH or DOPA decarboxylase in any of the three regions studied in PQ-treated mouse brain (data not shown).

### Differential expression patterns of α-synuclein observed in SN, FC and hippocampus of PQ (10 mg/kg b.w.)-treated mouse brain

**Figure 9:**
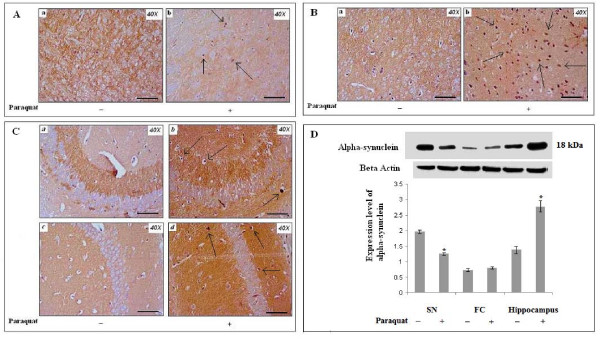
**Differential patterns of α-synuclein immunoreactivity after PQ (10 mg/kg b.w.) treatment**. α-Synuclein immunolocalization in SN, FC and hippocampus (A, B and C, respectively). (D) Densitometric analysis of western blot α-synuclein in SN, FC and hippocampus. **(A): **α-Synuclein immunopositivity was dense in neuritic areas of SN in controls (a). α-Synuclein immunopositivity decreased in neuritic areas, and a few pigmented nuclear structures (putative Lewy bodies) appeared (black arrows) in SN of PQ-treated animals (b). (**B): **α-Synuclein immunoreactivity in neuritic areas of FC in PQ-treated and control animals. Large numbers of pigmented nuclear structures (putative Lewy bodies) appeared (black arrows) in FC of PQ-treated brain (b), compared to vehicle-treated controls (a). **(C): N**on-nucleated areas (neuritic portions) but not nucleated areas, of hippocampus showed α-synuclein immunopositivity in both control and treated animals. Dense α-synuclein immunoreactivity appeared in non-nucleated areas of hippocampal regions CA3 and CA1 (b, d, respectively) in PQ-treated mouse brain compared to controls (a, c, respectively). A few pigmented deposits (putative Lewy bodies) were found in non-nucleated areas of hippocampal regions of PQ-treated animals, but not in respective controls. **(D): **Densitometric analysis of western blots indicate that expression levels of α-synuclein decreased significantly in SN, increased significantly in hippocampus and were unaltered in FC of PQ-treated animals, compared to controls. β-Actin was used as a reference control. Data are presented as mean ± SEM. (n = 3). Asterisks (*) represent significant differences, with p < 0.05 (Student's t-test) in densitometric analyses. Magnification of IHC images is 40× and the scale bars = 40µm.

Differential patterns of α-synuclein expression are one of the major markers of pesticide-induced neurodegeneration, especially in Parkinson's disease-like conditions. To asses the hypothesis that increased ROS levels followed by dopaminergic neurodegeneration in PQ-treated mouse brain are associated with α-synuclein status in brain, we evaluated α-synuclein immunolocalization and immunoreactivity in SN, FC and hippocampus in treated mice using immunohistochemistry and western blot. Immunoexpression of α-synuclein was decreased in SN of PQ-treated mouse brain (Figure 9A), whereas immunoexpression of α-synuclein did not show any significant changes in FC (Figure 9B). High levels of α-synuclein immunoreactivity were found in hippocampal areas (CA1 and CA3) of PQ-treated mouse brain compared to controls (Figure 9C). Some putative Lewy body-like structures with dense α-synuclein immunopositivity also appeared in all three regions of brain. Western blot analysis revealed that expression levels of α-synuclein decreased significantly in SN, with no change in FC and significantly increased expression in hippocampus (Figure 9D).

Supplementation with α-tocopherol did not result in any significant changes in status of α-synuclein in any of the three regions examined in PQ-treated mouse brain (data not shown).

### Changes of immunomodulatory molecules in PQ-treated mice brain

To assess whether differential expression patterns of α-synuclein in PQ-mediated neurotoxicity promotes inflammation or not, we evaluated expression levels of IL-1β and TNF-α in three different regions of brain.

#### Differential expression and localization of interleukin-1β (IL-1β) in SN, FC and hippocampus

**Figure 10:**
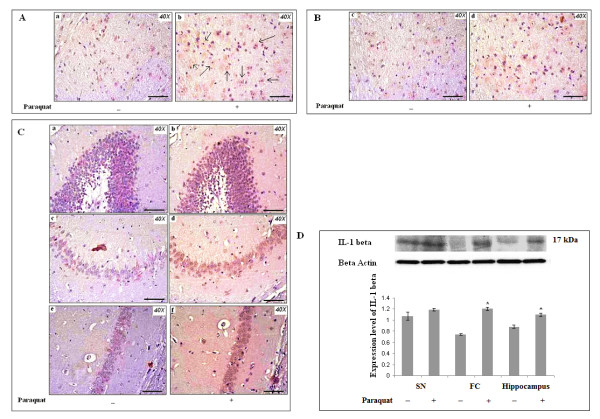
**Differential patterns of IL-1β immunoreactivity in brain after PQ (10 mg/kg b.w.) treatment**. IL-1β immunolocalization was assessed in SN, FC and hippocampus (A, B and C respectively). Densitometric analysis of western blots for IL-1β (D) was performed to assess expression levels for IL-1β in SN, FC and hippocampus. **(A): **IL-1β immunoreactivity is dispersed in SN of PQ-treated animals (b) compared to controls (a). Immunoreactive deposits are found in a distinct population of small, rounded cells (putative microglia, black arrows) rather than nucleated neuronal cells, in SN of PQ-treated animals, but not in controls. **(B): **A diffuse pattern of IL-1β immunoreactivity appeared in FC of PQ-treated animal (d) with more intense immunoreaction compared to controls (c). **(C): **Both nucleated and non-nucleated areas of hippocampus (dentate gyrus, CA3 and CA1; b, d, f respectively) of PQ-treated brain show more intense IL-1β immunoreactivity compared to controls (a, c and e respectively). Nucleated areas of hippocampus of PQ-treated mice showed dense immunoreaction compared to non-nucleated areas (b, d and f). **(D): **Densitometric analysis of western blots indicates that expression levels of IL-1β increased significantly both in FC and hippocampus without any change in SN of PQ-treated animals, compared to controls. β-Actin was used as a reference control in western blots. Data are presented as mean ± SEM (n = 3). Asterisks (*) represent significant differences at a level of p < 0.05 (Student's t-test) for densitometric analses. Magnification of IHC images is 40× and the scale bars = 40µm.

Differential expression patterns of the proinflammatory cytokine IL-1β in the three regions of brain were observed. Scattered and diffuse immunoreactivity for IL-1β increased in SN, FC and hippocampus in the PQ-treated groups compared to control groups (Figure 10A, B and 10C respectively). Immunoreactivity for IL-1β appeared outside of nucleated areas as patches. Some microglia-like cellular structures surrounded by dense immunoreactivity for IL-1β were observed in SN (Figure 10A). Western blot analysis showed that expression levels for IL-1β increased significantly in hippocampus and in FC during PQ treatment, without any significant changes in SN (Figure 10D). Supplementation with α-tocopherol did not produce any changes in status of expression levels for IL-1β in any of the three regions examined in PQ-treated mouse brain.

#### Tumor necrosis factor-α (TNF-α) is increased in SN, FC and hippocampus of PQ (10 mg/kg b.w.)-treated animals

**Figure 11:**
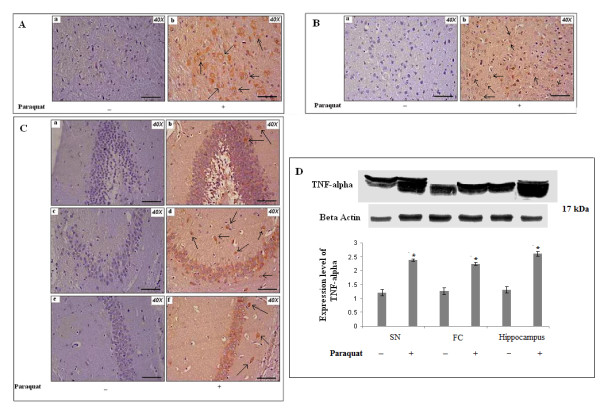
**PQ (10 mg/kg b.w.) treatment increases TNF-α immunoreactivity in brain**. TNF-α immunolocalization was assessed in SN, FC and hippocampus (A, B and C respectively). Densitometric analyses of western blots for TNF-α (D) were performed to assess expression levels of TNF-α in SN, FC and hippocampus. **(A): **TNF**-**α immunoreactivity was more intense in SN of PQ-treated animals (b) compared to controls (a). TNF-α immunopositivity was found in both neuritic areas and in large, oval-shaped cells (putative microglia-macrophages, black arrows) in SN of PQ-treated animals (b). **(B): **The FC of PQ-treated animals (b) showed more intense TNF**-**α immunoreactivity compared to controls (a). Immunoreaction appeared in neurites and in small round cells (putative microglia, black arrows) in FC of PQ-treated animals (b). **(C): **TNF-α immunoreactivity was found in both nucleated and non-nucleated areas of hippocampus, with more intense immunoreaction in hippocampal regions of PQ-treated animals (dentate gyrus, CA3 and CA1; b, d and f respectively). TNF-α immunopositive small round cells (putative microglia, black arrows) appeared in both nucleated and non-nucleated areas of hippocampus of PQ-treated animals (b, d and f). **(D): **Densitometric analyses of western blots indicate that TNF-α expression levels increased significantly in all three regions (SN, FC and hippocampus) of PQ-treated animals compared to controls. β-Actin was used as a reference control in western blots. Data are presented as mean ± SEM (n = 3). Asterisks (*) represent significant differences at a level of p < 0.05 (Student's t-test) for densitometric analyses. Magnification of IHC images is 40× and the scale bars = 40µm.

**Figure 12:**
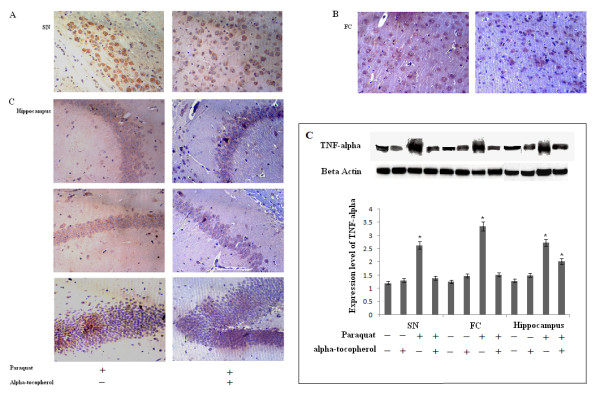
**Tocopherol supplementation reduces PQ (10 mg/kg b.w.)-induced TNF-α overexpression**. TNF-α immunolocalization in SN, FC and hippocampus after PQ treatment with or without tocopherol supplementation (A, B and C respectively). Densitometric analyses of western blots indicate that tocopherol supplementation reduced expression levels of TNF-α significantly in SN, hippocampus, and FC of PQ-treated animals compared to PQ-treated animals without tocopherol. **(A): **TNF-α immunoreactivity of oval-shaped cells (microglia-macrophages) appeared in SN after PQ treatment (a), and this was reduced by α-tocopherol (b). **(B): **TNF-α immunopositivity appeared in microglia-like cells in FC after PQ treatment (a), and this was reduced by tocopherol (b). **(C): **TNF-α immunoreactivity appeared in both nucleated and non-nucleated areas of hippocampal regions CA1, CA3, and dentate gyrus (a, c and e respectively) after PQ treatment (b, d and f respectively). PQ treatment followed by tocopherol reduced TNF-α immunoreactivity in nucleated and non-nucleated areas of hippocampus (b, d and f respectively). **(D): **Densitometric analyses of western blots indicate that PQ treatment increased TNF-α expression levels in SN, FC and hippocampus compared to respective saline-treated control. Expression of TNF-α in the three regions remained unaltered after PQ treatment followed by tocopherol, compared to controls. Tocopherol did not alter TNF-α expression compared to saline-treated controls. β-Actin was used as a reference control in western blots. Data are presented as mean ± SEM. (n = 3). Asterisks (*) represents significant differeneces at a level of p < 0.05 (Student's t-test) for densitometric analyses. Magnification of IHC images is 40× and the scale bars = 40µm.

We also evaluated another inflammation marker, TNF-α, in all three regions of PQ-treated mouse brain. Changes in expression patterns for TNF-α in all three experimental areas were assessed using immunohistochemistry and western blot. Immunoreactivity for TNF-α increased in all three regions of mouse brain treated with PQ compared to control groups (Figure 11A, B, C). Immunoreactivity of TNF-α appeared throughout the tissue sections, including both nucleated and non-nucleated portions, in all three experimental areas. However, this immunoreactivity was highest surrounding nucleated cells in SN and hippocampus. There were many microglia-like, large, rounded structures surrounded by dense TNF-α immunoreactivity that appeared particularly in SN and hippocampus (Figure 11A, C) in PQ-treated mice brain. Some small, rounded structures also appeared in FC but these were very few in number (Figure 11B) in treated brain. Apart from that unknown cellular structure, dispersed and high immunoreactivity for TNF-α was observed in nucleated areas of FC in PQ-treated mouse brain compared to controls (Figure 11B). Western blot analysis revealed significantly increased expression of TNF-α in all three regions of brain in treated animals (Figure 11D). Supplementation with α-tocopherol resulted in significant decreases in immunoexpression of TNF-α in all three regions of PQ-treated mouse brain compared to PQ-treated mouse brain. Immunoreactivity of microglia-like cells decreased in SN, FC and hippocampus of α-tocopherol-supplemented, PQ-treated groups compared to PQ-treated groups (Figure 12A, B and 12C). Western blot analysis revealed that α-tocopherol supplementation to PQ-treated mice decreased TNF-α expression levels significantly in all three regions of brain compared to PQ-treated mouse brain (Figure 12D).

### Differential expression patterns of Iba1 as a microglial marker and Mac 1 as a microglial activation marker

**Figure 13:**
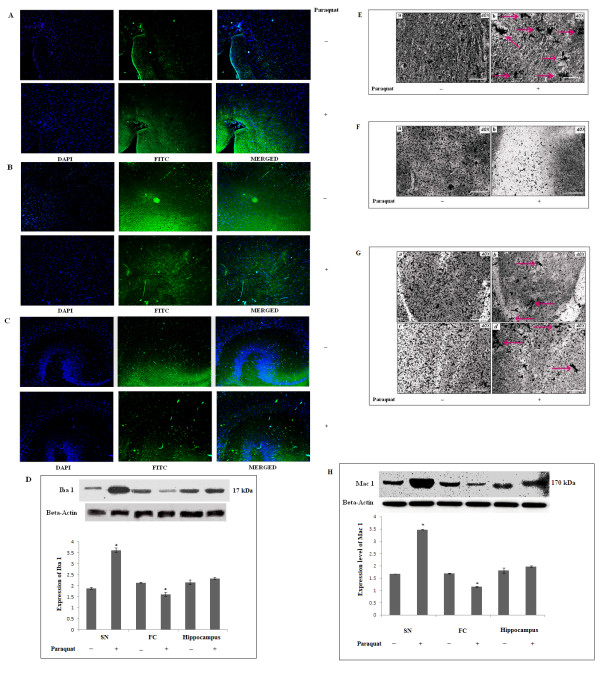
**Microglial activation after PQ (10 mg/kg b.w.) treatment**. Iba 1 immunolocalization (microglial marker) in SN, FC and hippocampus (A, B and C respectively; Weil and Davenport's method -- gray scale) in SN, FC and hippocampus (E, F and G respectively). Densitometric analyses of western blots for Iba 1 and Mac1 (microglial activation marker) to assess expression levels in SN, FC and hippocampus (D and H respectively): **(A) i**ntensity of Iba 1 immunofluorescence increased in SN of PQ-treated animals; **(B, C) **intensity of Iba 1 immunofluorescence decreased in FC (B) and hippocampus (C) of PQ-treated animals. **(D) **Densitometric analyses of western blots: Iba 1 expression increased significantly in SN, decreased in FC and remained unaltered in hippocampus of PQ-treated mice compared to vehicle-treated controls. **(E, F and G): **Silver staining revealed a reactive phenotype for microglia (red arrow), which appeared in SN as aggregated forms (b) in PQ-treated brain (E). Silver-impregnated microglia decreased in FC of PQ-treated animals (F). Aggregated forms of microglia appeared in hippocampus (G) of PQ-treated brain, but not controls. **(H) **Densitometric analyses of western blots indicate that Mac1expression increased significantly in SN, decreased significantly in FC and remained unchanged in hippocampus of PQ-treated animals. β-Actin was used as a reference control in western blots. Data are presented as mean ± SEM (n = 3). Asterisks (*) represent significant differences at a level of p < 0.05 (Student's t-test) for densitometric analyses. Magnification of immunofluorescence images is 10× and magnification of silver-stained images is 40×; the scale bars = 40 µm.

Microglial cells release immuno-inflammatory molecules during injury in the central nervous system (CNS). Increases in expression levels of IL-1β and TNF-α in all three experimental areas of PQ-treated mouse brain (as indicated in Figure 10 and 11) indicate that neuroinflammation is involved in PQ-mediated, ROS-induced dopaminergic neurodegeneration. There were also microglia-like, large cellular structures in SN and hippocampus of PQ-treated brain. Therefore, to evaluate involvement of microglial cells in PQ-mediated neurotoxicity, the appearance of microglial cells and expression levels of microglial markers were assessed by immunofluorescence. We observed that Iba1 immunoreactivity and expression levels significantly increased in SN, but significantly decreased in FC with no significant change in hippocampus, of PQ-treated animal compared controls (Figure 13A, B, C and 13D). To assess whether microglial cells appeared activated or not, we evaluated the microglial activation marker Mac1. Western blot analysis revealed that there was a significant increment in Mac 1 expression in SN, but this was decreased significantly in FC and no changes were observed in hippocampus of PQ-treated brain compared controls (Figure 13D). Supplementation with α-tocopherol in PQ-treated animals did not reproduce any change in expression levels of Iba1 or of Mac 1 in any of the three regions of brain examined, compared to PQ-treated mouse brain (data not shown).

### Histological changes in microglial cells

To further assess microglia, we used Weil and Davenport's method for microglia-specific silver staining [23], applied to determine activation of microglial cells or microgliosis, as a priming event of neuroinflammation (Figure 13). Microscopical observation revealed numerous aggregations of microglial cells in SN, compared to controls, and also changes in microglial morphology (Figure 13E). Microglia were less apparent in FC of PQ-treated brain compared to control (Figure 13F), and this likely indicates microglial degeneration or microglial migration. In hippocampus, aggregated microglial cells were present in PQ-treated brain compared to control mouse brain (Figure 13G).

## Discussion

The cellular and molecular mechanisms underlying PQ-induced neurodegeneration are unclear. Several studies have indicated that PQ toxicity causes dopaminergic neuronal cell loss in SN and expression of α-synuclein in SN as well as in FC through formation of superoxide radicals. In this study, PQ-treated animals showed several symptoms including impairment of motor performances, which developed after two weeks of treatment. These physiological changes are indicative of PQ-mediated neurotoxicity. Dopaminergic neurons are more vulnerable to PQ-induced oxidative stress than are other neuronal populations because they are ill equipped to endure oxidative stress [37,38]and because PQ promotes dopaminergic neuronal death via a c-Jun-N-terminal kinase 3 (JNK3)-mediated cell signaling pathway [39]. SN is rich in dopaminergic cells. Therefore, during PQ exposure, SN is the most affected region of brain.

The hippocampal formation receives projections from the midbrain's dopaminergic cell groups and contains mRNA for dopamine receptors [40], and FC receives output from SN via the thalamus [41]. Previous studies have shown that PQ deposition is found in hippocampus as well as in FC [42,43]. Therefore, apart from SN, PQ-mediated mitochondrial injury by oxidative stress has a toxic influence on hippocampus and FC of mice [18]. Therefore, along with SN, FC and hippocampus were major areas of concern during PQ-mediated neurotoxicity in the present study.

Multiple but sufficiently low doses of PQ are well tolerated by peripheral organs in rats without apparent oxidative stress [44]. In the present study, ROS levels increased (as reflected by high activity of ROS-scavenging enzymes) significantly in three regions of brain with PQ doses of 10 mg/kg b.w. or greater. However, ROS levels in peripheral organs increased significantly with all lethal doses (20 mg/kg b.w. to 80 mg/kg b.w.) but not with a sublethal dose (10 mg/kg b.w.) in our treatment regimen. Therefore, in our animal model, impaired motor function appeared to be due to PQ-induced, brain-specific ROS generation, which promotes neurodegeneration, but not due to ROS generation in peripheral organs. Hence, peripheral levels of ROS at a PQ dose of 10 mg/kg b.w. may not be sufficient to increase ROS levels in brain. We may consider that the rise in ROS levels in brain with a PQ dose of 10 mg/kg b.w. is a local effect, and that ROS may be involved in alteration of other parameters in brain. PQ needs time to cross the blood-brain barrier to reach brain tissue and generate ROS generation. As with other, higher, lethal doses, the availability of PQ in brain may not be sufficient to produce maximum toxicity. Such higher doses of PQ may produce acute peripheral toxicity that causes more severe peripheral tissue (e.g., lung, kidney, etc.) damage (Bhattacharyya, unpublished) and our cell counting data support this idea. Numbers of dopaminergic cells decreased most significantly in SN at a sublethal dose of PQ (10 mg/kg b.w.) compared to other lower or lethal doses and to controls. Therefore, we consider a dose of 10 mg/kg b.w. PQ as a sublethal dose that may produce high levels of ROS locally in brain. α-Tocopherol supplementation decreased ROS levels in all three regions of brain.

Increased ROS levels induced differential changes in cellular morphology in all three regions of brain in the present study. Pyknotic nuclei appeared in SN, in FC and in hippocampus of the treatment group, indicating that, apart from SN, hippocampus and FC are also affected by PQ-mediated neurotoxicity. Many Lewy body-like structures in FC indicate that there might be involvement of differential α-synuclein expression level not only in FC but also in SN and hippocampus. PQ exposure induced the formation of α-synuclein-containing deposits. This effect, however, was seen in both control and α-synuclein-overexpressing mice, thus suggesting that neuroprotection is not a mere consequence of lack of protein deposition. α-Synuclein itself may possess properties that counteract toxic injury, and its expression could affect specific stress signaling pathways linked to neuronal survival. For example, Hashimoto and colleagues [45]have suggested that α-synuclein expression can confer resistance to *in vitro *hydrogen peroxide toxicity via inactivation of c-Jun N-terminal kinase, a member of the mitogen-activated protein kinase family. This indicates that α-synuclein overexpression protects against Paraquat-induced neurodegeneration. α-Synuclein is not only a component of Lewy bodies and synapses but also of axons, and aggregated α-synuclein might interfere with axonal transport and lead to cell death [46]. Microscopical observations show that increased immunoreactivity for α-synuclein is predominant in FC of PQ-treated brain in our studies. However, α-synuclein protein expression levels do not change significantly in FC of PQ-treated mouse brain. Expression of α-synuclein did increase significantly in hippocampus, whereas expression levels of α-synuclein decreased in SN of our treated group. As in SN, cell death was more pronounced, suggesting that decreased α-synuclein levels indicate earlier degeneration in SN than in the other two regions of brain. In hippocampus, for example, α-synuclein levels increased to protect neuronal cells. Previous reports have stated that methylation of human α-synuclein gene intron 1 decreases that gene's expression, while inhibition of DNA methylation activates α-synuclein gene expression. On the other hand, extensive neuronal expression of α-synuclein and disruption of α-synuclein function or abnormal aggregation may have similarly widespread consequences [47]. Therefore, the reduced expression of α-synuclein in SN, increased expression in hippocampus, and aggregated forms in FC found in our present study might correlate with the α-synuclein gene polymorphism associated with PQ-mediated neurotoxicity in this mouse model and the differential time frames necessary to initiate neurodegeneration in these different regions.

In relation to the hypothesis that differential α-synuclein expression may modulate TH expression levels, we evaluated TH-positive neuronal vulnerability in all three regions of PQ-treated mouse brain. We observed that expression levels of TH decreased in all three regions of PQ-treated mouse brain. The reduction of TH expression may indicate a reordering of protein biosynthesis favoring production of protein required for axonal regeneration at the expense of those involved in neurotransmission [48]. The most interesting observation is that the distribution of TH immunoreactivity changes from nucleated areas to non-nucleated areas in hippocampus and FC, but not in SN. Hence, we may predict that TH, which is present in axons (non-nucleated areas) shows greater immunoreactivity in non-nucleated areas compared to nucleated areas in hippocampus and FC under our treatment conditions. The presence of pyknotic nuclei in SN may explain in part the total reduction of TH in SN. However, the exact cause of such differential patterns of TH expression in three brain regions under our treatment conditions needs further investigation. Dopaminergic immunoreactivity decreased primarily in SN and FC, while in hippocampus no significant changes were observed. At the same time, there might be neuronal compensatory mechanisms involved to regenerate new dopaminergic neurons such that overall levels of DOPA decarboxylase and FOX3 expression level do not change.

In the present study, differential expression patterns of α-synuclein and TH due to PQ treatment elicited high expression levels of proinflammatory cytokines such as TNF-α in the three regions of mouse brain, and of IL-1β in FC and hippocampus of mouse brain. There might be involvement of activated microglial cells in promoting neuroinflammation. Microglial cells in a resting state continuously maintain homoeostatic activity in the CNS, and in a fully activated phagocytic state microglial cells scavenge neurotoxins, remove dying cells and cellular debris, and secrete trophic factors that promote neuronal survival, reorganization of neuronal circuits and repair [49,50]. Insufficient clearance by microglia is prevalent in several neurodegenerative diseases and in normal ageing [51]. Over-activation of microglia may cause alterations in immunophenotypic expression and inflammatory profile (promoting microglia senescence), and that condition may switch microglial function from neuroprotective to neurotoxic effects [52]. Increased expression of Mac1 (microglial activation marker) in SN indicates chronic neuroinflammation. However decreased expression of Iba1 (a microglial marker) and Mac 1 with increased cytokine levels in FC might reflect the peripheral supply of cytokines without local production by microglial cells in brain.

Long-standing activation of microglia during chronic neuroinflammation causes sustained release of inflammatory mediators that promote activation of additional microglial proliferation, and further release of inflammatory factors [53]. In search of involvement of microglial cells in PQ-mediated neurotoxicity; we have found aggregated microglial cells in SN of PQ-treated mouse brain, while microglia-specific staining is less positive in FC. Although there are aggregated microglial cells in hippocampus of treated mouse brain, microglial cell-specific staining decreases compared to controls in hippocampus. At this point it is not clear whether microglial cells degenerate or migrate to other areas with pathogenic lesioning. Chemokines regulate rapid migration of microglia to injury sites in CNS and amplify neuroinflammation [54]. If microglial cells degenerate, then this degeneration presumably relates to a failure of neuroprotective functions and subsequent contributions to neurodegeneration [55]. Recent studies indicate that death of microglial cells may occur as a consequence of overproduction of immuno-inflammatory molecules along with production of anti-inflammatory molecules such as IL-13, activation of *Fas*-mediated apoptotic signaling, and/or toxins produced by over-activated microglial cells themselves [56,57]. Although microglial phenotypic shifting, as seen in our study, occurs in frontal cortex and hippocampus, we found significant high levels of inflammatory molecules in all three regions of brain. Proinflammatory cytokines released from over-activated microglia may act on the endothelium of BBB cells to stimulate upregulation of adhesion molecules for passage of T cells and monocytes that then go on to release more cytokines. This indicates that chronic inflammation may cause an increase in permeability of the BBB [58-61]. From this point of view, we may predict that the high levels of pro-inflammatory/inflammatory molecules in the three regions of brain found in our present study may participate in increasing a peripheral supply of inflammatory responses to those areas of brain. Further investigation is needed to explore these phenomena. As α-tocopherol supplementation decreased TNF-α levels in all three regions of PQ-treated mouse brain, ROS-induced TNF-α production might initiate neuroinflammation with or without involvement of microglial cells during PQ treatment. Other parameters, such as α-synuclein, dopaminergic neuronal status and microglial status, were not altered with α-tocopherol supplementation in PQ-treated brain. To maintain the same time frame of sacrifice as PQ-treated mice (day 7 after the last dose of PQ), we supplemented different sets of PQ-treated mice with five doses of α-tocopherol only. Therefore, further study is needed to evaluate the effects of α-tocopherol supplementation in PQ-treated mice brain for prolonged time spans.

## Conclusion

We conclude that PQ-mediated neurotoxicity acts mainly via ROS generation with involvement of differential expression patterns for α-synuclein in SN, FC and hippocampus of mouse brain. Neuroinflammation took place with or without involvement of microglial activation during PQ treatment and dopaminergic neuronal status changed differentially. Involvement of these differential changes might indicate separate signaling phenomena and different time frames for initiation of neurodegeneration in SN, FC and hippocampus of mouse brain due to PQ treatment. The exact cause of such changes and their correlation need further study.

## List of abbreviations

PD: Parkinson's disease; SN: Substantia nigra; FC: Frontal cortex; PQ: Paraquat; TH: Tyrosine hydroxylase; ROS: Reactive Oxygen Species; GST: Glutathione-S-Transferase; SOD: Superoxide dismutase; BBB: Blood brain barrier; Mac1: Macrophage antigen complex-1; PBS: Phosphate-buffered saline; MPP+: 1-methyl-4-phenylpyridinium; MPTP: 1-methyl-4-phenyl-1,2,3,6-tetrahydropyridine; TNF-α: Tumor necrosis factor-alpha, IL-1β: Interleukin-1beta; b.w.: Body weight.

## Competing interests

The authors declare that they have no competing interests.

## Authors' contributions

SM, NC and AB developed the concept, designed the experiments, and contributed in the data analysis and writing of manuscript. SM carried out all experiments. NC assembled and interpreted all results. AB evaluated and coordinated the whole work. All authors read and approved the final manuscript.
